# EZH2 Inhibition Ameliorates Transverse Aortic Constriction-Induced Pulmonary Arterial Hypertension in Mice

**DOI:** 10.1155/2018/9174926

**Published:** 2018-02-28

**Authors:** Zhan-Li Shi, Kun Fang, Zhi-Hui Li, Dan-Hong Ren, Jia-Ying Zhang, Jing Sun

**Affiliations:** Department of Intensive Care Unit, Hangzhou Red Cross Hospital/Zhejiang Chinese Medicine, Western Medicine Integrated Hospital, Hangzhou 310003, China

## Abstract

**Background:**

EPZ005687 is a selective inhibiter of methyltransferase EZH2. In this article, we investigated the protective role and mechanism of EPZ005687 in transverse aortic constriction-induced pulmonary arterial hypertension in mice.

**Methods:**

We assigned 15 (6–8 weeks old) male balb/c mice to 3 groups randomly: Sham control + DMSO group, TAC + DMSO group, and TAC + EPZ005687 group (10 mg kg^−1^, once a week for 4 weeks). On day 28 following TAC operation, the right ventricular systolic blood pressure (RVSBP) was measured, and lung tissues were collected for laboratory examinations (DHE, Western blot, real-time PCR, and ChIP).

**Results:**

Murine PAH model was successfully created by TAC operation as evidenced by increased RVSBP and hypertrophic right ventricle. Compared with the sham control, TAC-induced PAH markedly upregulated the expression of EZH2 and ROS deposition in lungs in PAH mice. The inhibiter of methyltransferase EZH2, EPZ005687 significantly inhibits the development of TAC-induced PAH in an EZH2-SOD1-ROS dependent manner.

**Conclusion:**

Our data identified that EZH2 serves a fundamental role in TAC-induced PAH, and administration of EPZ005687 might represent a novel therapeutic target for the treatment of TAC-induced PAH.

## 1. Introduction

There is a growing body of literature that recognizes the end stage of left ventricle heart failure often leading to pulmonary artery hypertension (PAH) and right ventricle heart failure [1–3]. Abundant evidences have investigated the mechanism and therapeutic strategy of heart failure (HF) [4–6]. Yet, there is poorly published data concerning the PAH induced by HF. Recent evidences suggest that the accumulation of reactive oxygen species (ROS) may contribute to the pathogenesis and development of PAH [7, 8]. Furthermore, antioxidant treatments delay the development of PAH, intimating that pulmonary oxidative stress mediates the development of PAH [1, 8].

Epigenetic gene regulations are vital to cardiovascular diseases (CVDs) [9, 10]. Various histone modifications on the promoter involved in genetic expression pattern, activation, or repression; histone H3 lysine 27 trimethylation (H3K27me3) exerts a key role in gene repression [11]. Evidences suggest that H3K27me3 is an important factor for cardiac hypertrophy [12] and generation of ROS [13, 14]. Suppressor of enhancer of zeste homolog 2 (EZH2) is a methyltransferase responsible for laying down the H3K27me3 mark on the chromatin [15, 16]. EZH2 was initially discovered as crucial regulator centrally involved in the regional organization of homeotic gene expression implicated in the assembly of chromatin domains and the regulation of chromatin of repressive protein complexes in chromatin [17]. Although EZH2 was reported to inhibit cardiac hypertrophy by recruiting H3K27me3 on prohypertrophic genes like ANP, BNP [18], and CaMKII [19], it remains obscure how EZH2 may contribute to the pathogenesis of TAC-induced PAH.

The aim of this essay is to discuss the relationship between H3K27me3 modification statuses in TAC-induced PAH. EPZ005687 is a selective inhibitor of the lysine-specific histone methyltransferase EZH2 [20]. Therefore, another objective of this study was to determine whether the administration of EPZ005687 attenuated the progression of PAH induced by TAC. Here, we report that upregulation of EZH2 accompanies SOD1 downregulation in the lung during TAC-induced PAH. Pharmaceutical inhibition of EZH2 delays progression of TAC-induced PAH in mice through inhibiting oxidative stress in lung.

## 2. Methods

### 2.1. Animal Preparation

This study was approved by the Animal Ethics Committee of Zhejiang Chinese Medicine and Western Medicine Integrated Hospital. Balb/c mice (Shanghai Biomodel Organism Science & Technology Development Co., Ltd.) were used for the described experiment. Balb/c mice (6–8 weeks) were assigned randomly to three experimental groups: (a) sham control group, injected peritoneally with DMSO for 4 weeks once a week; (b) TAC group, injected peritoneally with DMSO for 4 weeks once a week; and (c) TAC group, injected peritoneally with EPZ005687 (10 mg kg^−1^) for 4 weeks once a week. The injection was performed at 1, 8, 15, and 22 days post-TAC or sham operation.

### 2.2. Transverse Aortic Constriction (TAC) Procedure

In brief, 6–8 weeks old male mice (body weight ranging from 22 to 25 g) were operated on using a TAC operation. Before the operation, the mice were anaesthetized with a mixture of ketamine (120 mg kg^−1^) and xylazine (6 mg kg^−1^) (i.p.). A horizontal incision 5 mm in length is made at the level of the suprasternal notch to allow direct access to the transverse aorta without damaging the pleural space. Aortic constriction is performed by ligating the aorta between the right innominate artery and the left carotid artery over a 27-gauge needle using 6-0 silk sutures for approximately 70% aortic constriction. After aorta ligation, the needle is then quickly removed; leave the constriction in place and then the thoracic cavity was closed. Successful constriction of the aorta was identified and confirmed by Doppler echocardiogram. Sham-operated animals underwent the same surgical procedure without partial aorta ligation [4].

### 2.3. RV Pressure Measurements

At the end of the study protocol, open-chest RV catheterization was performed to detect RV Pressure under general anesthesia in all animals (isoflurane 2.0%) as described. The right ventricle was approached via a lateral right thoracotomy through the fifth intercostal space. RV pressures were recorded by the use of a 1.2-F pressure catheter (Scisense Inc., Ontario, Canada) [21].

### 2.4. Histological Analysis

Heart weight (HW), lung weight (LW), and body weight (BW) were measured. For ROS detection, frozen lung sections (5 *μ*m) were stained with DHE (2 *μ*mol/l Selleck) in a light-protected humidified chamber at 37°C for 30 min. The slides were visualized by confocal fluorescence microscopy (Zeiss).

### 2.5. Western Blot

Whole lysates were obtained with RIPA buffer (50 mM Tris pH 7.4, 150 mM NaCl, 1% Triton X-100) with freshly added protease inhibitor cocktail tablet (Roche); the protein concentrations were measured by using the Pierce® BCA Protein Assay Kit (23225, Thermo Fisher Scientific, Waltham, MA, USA). The nitrocellulose membranes (1620112, Bio-Rad) were subsequently blocked in TBST containing 5% skimmed milk powder for 90 minutes at room temperature and incubated with anti-EZH2 (Peninsula Laboratories Inc.), anti-SOD1 (Santa Cruz Biotechnology), anti–nitrotyrosine (Santa Cruz Biotechnology), and anti-*β*-actin (Sigma) overnight at 4°C. On following day, the membranes were incubated with secondary antibodies, respectively, and the signals were visualized with a SignalFire(tm) ECL Reagent (6883S, Cell Signaling Technology, USA).

### 2.6. RNA Extraction and Quantitative Real-Time PCR

Total RNA was isolated using TRIzol (Invitrogen) according to the manufacturer's instructions. cDNA was synthesized from 1 *μ*g of RNA with the One Step RT-PCR Kit (Takara). Quantitative PCR was processed as described before [22]. Primer pairs used for real-time PCR are as follows: SOD1, forward 5′-ACTGGTGGTCCATGAAAAAGC-3′ and reverse 5′-AACGACTTCCAGCGTTTCCT-3′; [23] EZH2, forward 5′-TGGACCACAGTGTTACCAGCA-3′ [24] and reverse 5′-TGGGCGTTTAGGTGGTGTCT-3′; GAPDH, forward 5′-TGGTTCACACCCATCACAAACA-3′ and reverse 5′-GGTGAAGGTCGGTGTGAACGG-3′. The relative amount of each gene in each sample was estimated by the ΔΔCT method. Results were normalized to Gapdh RNA level.

### 2.7. Chromatin Immunoprecipitation, ChIP

ChIP assays were performed as previously described [25]. In brief, Chromatin was cross-linked with 1% formaldehyde. Cells were incubated in lysis buffer (150 mM NaCl, 25 mM Tris pH 7.5, 1% Triton X-100, 0.1% SDS, 0.5% deoxycholate) supplemented with protease inhibitor tablet. DNA was fragmented into ∼500 bp pieces using a Branson 250 sonicator. Aliquots of lysates containing 100 *μ*g of protein were used for each immunoprecipitation reaction with anti-LSD1 (Abcam) and anti-trimethylated H3K27 (Abcam). Precipitated genomic DNA was amplified by real-time PCR with the following primers: Gapdh promoter region, 5′-ATCACTGCCACCCAGAAGACTGTGGA-3′, and 5′-CTCATACCAGGAAATGAGCTTGACAAA-3′; Sod1 promoter region, 5′-AATAGCGACTTTCCCAGCTC-3′, and 5′-AAACGAAGGTGCAAAACGAG-3′.

### 2.7. Statistical Analysis

All data are presented as the mean ± standard error. Data of two groups were compared with unpaired *t*-test. One-way analysis of variance with post hoc Scheffe analyses was performed using an SPSS package. Grouping was performed in a randomized manner.

## 3. Results

### 3.1. Pulmonary Arterial Hypertension Induced by TAC Simulates EZH2 Expression

**Figure 1:**
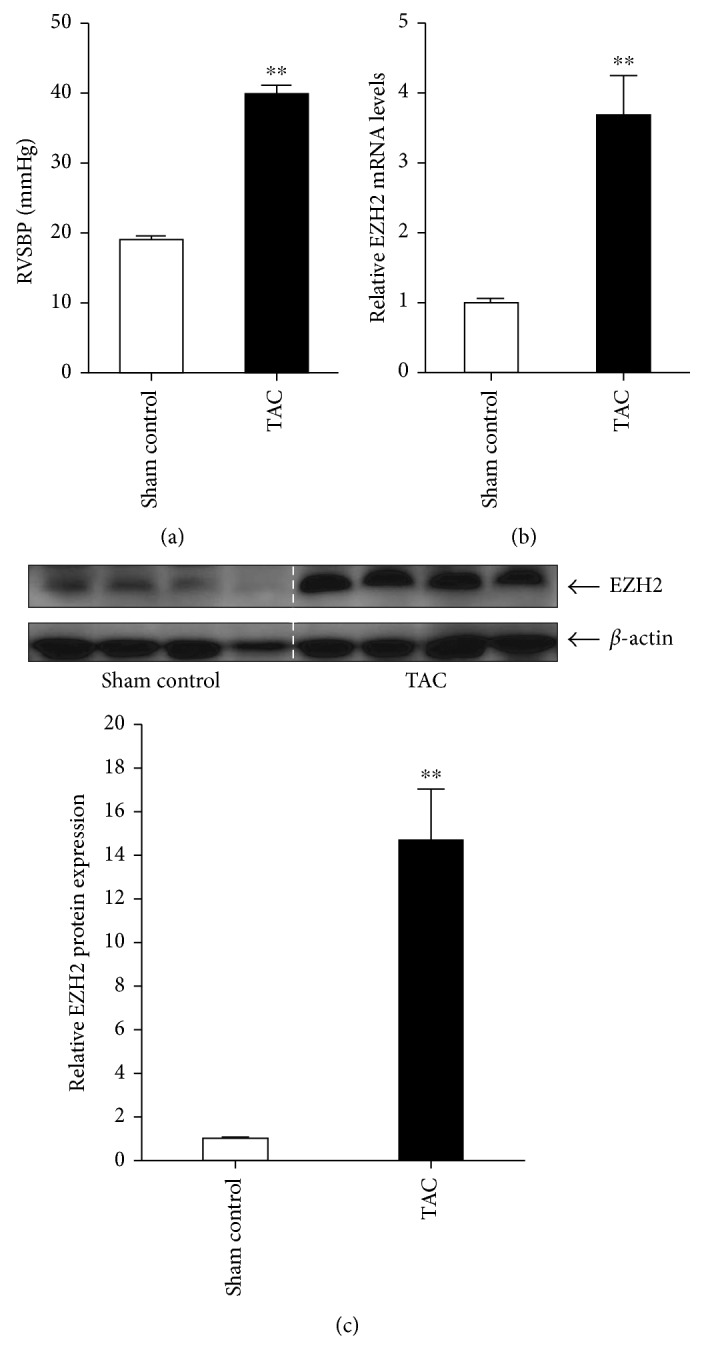
Expression of EZH2 is upregulated in lungs in TAC-induced PAH mice. (a) Right ventricular systolic blood pressure (RVSBP) at day 28 after TAC procedure (*n* = 4 for each group, ^∗∗^*P* < 0.001 versus sham control). Relative mRNA (b) and protein (c) levels of EZH2 in lungs in sham control mice or TAC mice on day 28 following TAC procedure (*n* = 4 for each group, ^∗∗^*P* < 0.001 versus sham control).

First of all, whether the PAH murine model was successfully induced by TAC surgery should be verified. Additional 8 balb/c mice were used for sham control and TAC procedure without any treatment. As was shown Figure 1(a), on day 28, TAC group mice developed PAH as evidenced by the increase in right ventricular systolic pressure (RVSBP), an index of pulmonary arterial systolic blood pressure (PABP). To investigate the role of EZH2 in the progression of PAH induced by TAC, we measured the EZH2 levels in lung tissues in mice. Western blot analysis and quantitative real-time PCR demonstrated that TAC-induced PAH resulted in a significant induction of EZH2 in PAH lungs as compared with sham control lungs (Figures 1(b) and 1(c)). These results suggest that PAH is successfully induced by TAC, and expression of EZH2 in lungs is notably activated by TAC-induced PAH, which indicates that EZH2 may be involved in TAC-induced PAH.

### 3.2. EZH2 Inhibition Protects against TAC-Induced PAH In Vivo

**Figure 2:**
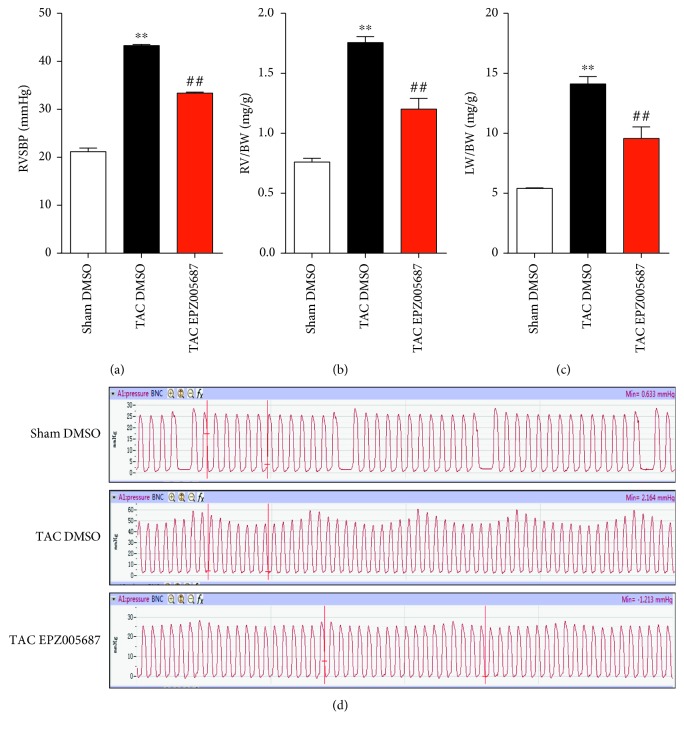
Inhibition of EZH2 with EPZ005687 attenuates TAC-induced PAH. (a) Right ventricular systolic blood pressure (RVSBP) at day 28 after TAC procedure. (b) Ratios of LV weight to body weight. (c) Ratios of lung weight to body weight (*n* = 5 for each group, ^∗∗^*P* < 0.001 versus sham control, ^##^*P* < 0.001 versus TAC DMSO). (d) RVSBP and the effect of EPZ005687 on pulmonary vessel tension.

To assess the biological impact of EZH2 on the development of pulmonary hypertension, we first measured the RVSP as an indicator of pulmonary artery pressure in spontaneously breathing mice, following sham control and TAC operation, EPZ005687 or DMSO was injected peritoneally as described in methods. On day 28 after TAC, the RVSBP was markedly higher in the TAC group than in the sham control group (Figures 2(a) and 2(d)). Besides, as shown in Figures 2(b) and 2(c), ratios of RV and lung weight to body weight were significantly increased 2.1-fold and 2.6-fold in TAC mice compared to sham control mice, respectively. EPZ005687 treatment significantly alleviated the further increase of RVSBP, ratios of LV and lung weight to body weight, respectively. These findings suggest that EPZ005687 treatment might have a prominent role in protecting against TAC-induced PAH and RV hypertrophy in vivo.

### 3.3. EZH2 Inhibition Regulates Oxidative Reactions in Lungs

**Figure 3:**
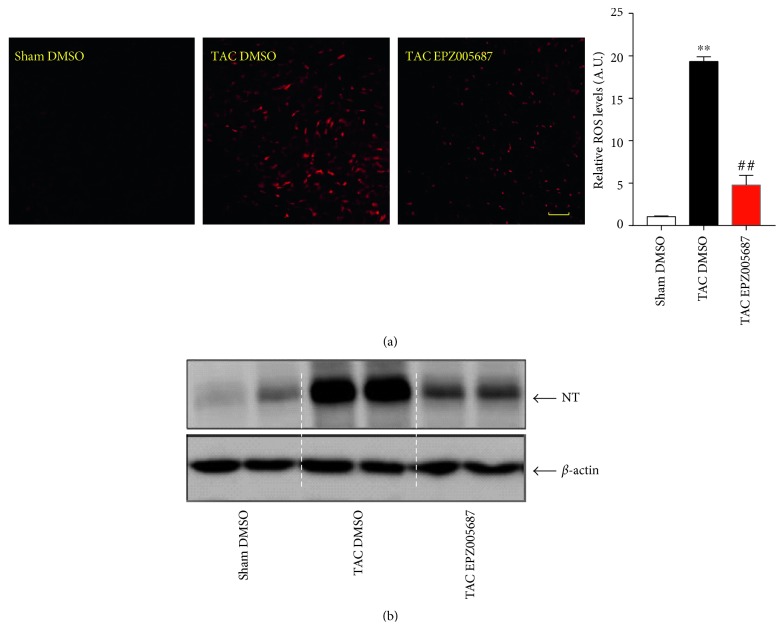
EPZ005687 treatment inhibits oxidative reactions in lungs in TAC-induced PAH mice. (a) Lung tissues ROS levels were evaluated by DHE staining (scale bar, 50 *μ*m, *n* = 5 for each group, ^∗∗^*P* < 0.001 versus sham control, ^##^*P* < 0.001 versus TAC DMSO). (b) The expression of nitrotyrosine in lung lysate was detected by western blot.

ROS are important parts of physiological and pathological processes. Recently, ROS are thought to be signaling molecules to mediate specific cellular responses in the vasculature [26]. Moreover, evidence shows that the accumulation of ROS contributes to the development of PAH [7, 8]. To investigate the role of ROS in our TAC-induced PAH model, DHE fluorescence probe was utilized to detect ROS production in murine frozen lung sections.  Figure 3(a) showed that EZH2 silencing prevented the induction of ROS production by TAC-induced PAH from 18.3 fold to 4.8 fold compared with sham control. As anticipated, the increase of lung nitrotyrosine caused by TAC-induced PAH was significant inhibited by EPZ005687 treatment (Figure 3(b)). These findings suggest that inhibition of EZH2 reduces oxidative reaction in lungs caused by TAC-induced PAH.

### 3.4. EZH2 Suppresses SOD1 Transexpression in Lung in TAC-Induced PAH Mice

**Figure 4:**
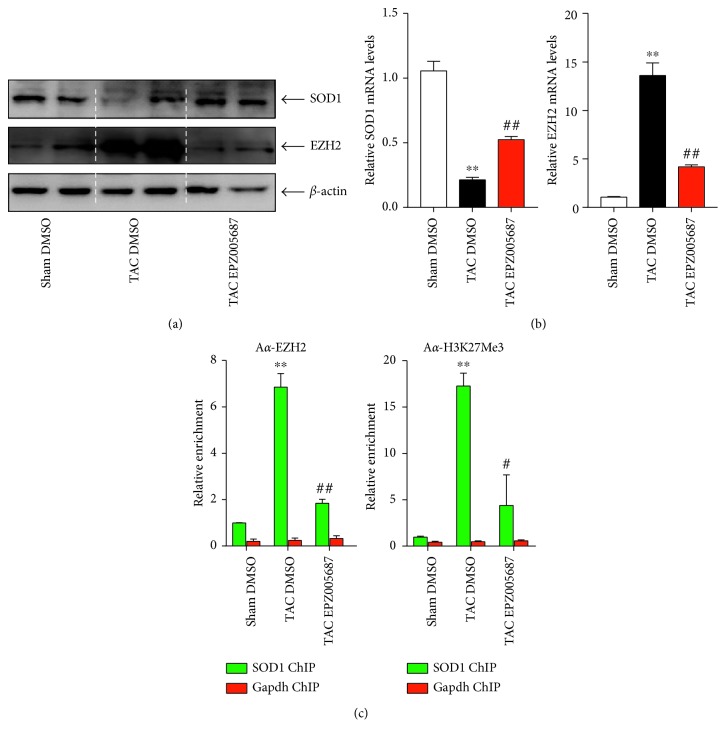
EZH2 regulates TAC-induced PAH by targeting SOD1. Expression levels of SOD1 and EZH2 were measured by (a) Western blot and (b) qPCR (*n* = 5 for each group, ^∗∗^*P* < 0.001 versus sham control, ^##^*P* < 0.001 versus TAC DMSO). (c) ChIP assays were performed with anti-EZH2 and anti-H3K27Me3 using lung tissues in indicated mice (*n* = 5 for each group, ^∗∗^*P* < 0.001 versus sham control, ^##^*P* < 0.001, ^#^*p* < 0.01 versus TAC DMSO).

SOD1 is responsible for destroying free superoxide radicals in the body. In accordance with the oxidative reaction in lungs, the expression of SOD1 was significantly downregulated and the expression of EZH2 was upregulated in TAC + DMSO group as compared with sham control, respectively (Figures 4(a) and 4(b)). Due to the negative correlation between EZH2 and SOD1 expression, and next, we tackled the relationship between EZH2 and SOD1. Interestingly, we observed EPZ005687 treatment could inhibit the expression of EZH2 and reverse the decreased expression of SOD1 (Figures 4(a) and 4(b)). Thus, we hypothesized that EZH2 might promote TAC-induced PAH by targeting SOD1. Chromatin immunoprecipitation (ChIP) assay showed that EZH2 and trimethylated histone H3K27 (H3K27Me3) accumulated on the SOD1 promoter but not the Gapdh promoter in TAC + DMSO group. EPZ005687 treatment was used to validate the role of EZH2 in SOD1 transrepression. Indeed, EPZ005687 treatment could reverse the increased accumulation of EZH2 and H3K27Me3 on SOD1 promoter (Figure 4(c)) and increase SOD1 expression in lung in TAC-induced PAH mice (Figures 4(a) and 4(b)). Combined, these data suggest that EZH2 regulates intracellular ROS levels in response to TAC-induced PAH stimulation through repressing SOD1 transcription.

## 4. Discussion

In this study, we investigated epigenetic treatment impact of EPZ005687 on TAC-induced PAH. At the end stage, left ventricle heart failure always leads to right ventricle heart failure, pulmonary remodeling, and pulmonary artery hypertension, which cause heavy economic and healthy burdens worldwide [1]. In this essay, we identified that the specific EZH2 inhibitor EPZ005687 attenuates TAC-induced PAH in an ROS-dependent manner through influencing histone H3K27 trimethylation on the SOD1 promoter.

There are abundant and consistent data for increased levels of ROS in patients and animal models of PAH [27, 28]. Notwithstanding, there is much diversity of research about the mechanisms that are responsible for the increased ROS, very few studies have investigated the influence of histone methylation modifications on ROS generation in PAH.

Histone methylation modifications play vital roles in gene expression.

Different histone methyltransferases are specific for the lysine residue they modify. On histone H3, for example, SET1, Ash2, WDR5, catalyze methylation of H3K4 in mammalian cells [5, 10, 22, 25], H3K4, H3K36, and H3K79 methylation generally represent gene activation. SUV39-h1 and SUV39-h2 histone methyltransferases that catalyze methylation of H3K9 [9], G9a [29], and EZH2 [30] are responsible for methylation of H3K27, whereas methylation on histones H3K9 and H3K27 is usually associated with gene repression.

Polycomb repressive complex 2 (PRC2) catalyze methylation of H3K27 and EZH2 is the catalytic subunit of the PRC2 complex [31]. EZH2-mediated methylation of lysine 27 on histone on target gene promoter suppresses target gene transexpression [12]. In the present research, we found that the expression of EZH2 was upregulated parallel with the accumulation of ROS and nitrotyrosine in lungs in PAH mice (Figures 3 and 4(a) and 4(b)). In addition, we also found that H3K27Me3 accumulation on the SOD1 promoter, likely mediated by EZH2, might be responsible for SOD1 transrepression in the lungs following TAC-induced PAH. EPZ005687 has been previously reported acting as an anticancer drug [32, 33] and attenuating osteoarthritis progression [34]. The present research broadens the scope of pharmacological applications of EPZ005687.

This study has some limitations. First, in the present study, RVSBP was evaluated at day 28 after TAC, by which time plenty of leukocytes would have infiltrated the lungs was extremely serious [35]. The leukocyte infiltration cannot be ignored when we try to interpret the current dataset, especially given the fact that EPZ005687 treatment was a systemic EZH2 inhibition mouse model. In view of the fact that the targeted cell type which could cause adverse oxidative reaction was not confirmed in the present study. It invites the question as to whether EZH2 inhibition in leukocytes could suppress ROS generation and, if so, via what mechanism(s). These issues need further investigation preferably with lineage-specific SUV39H knockout mice. Second, to create a severe heart failure for the following PAH progression, some of mice in TAC group did not survive at the end of experiment. Third, because left heart failure-induced PAH caused other lung injuries like pulmonary edema, the results of the present study could not identify the other injuries caused by TAC. Fourth, although we provide a single transcription event, mediated by EZH2-SOD1-ROS axis, which may be linked to TAC-induced PAH, there lacks a clear delineation as to how EZH2 coordinates genome-wide transcription to manipulate this process. Fifth, 10 mg kg^−1^ (i.p.) for 4 times for four consecutive days might be sufficient to suppress the expression EZH2 in the present study; strict toxicity and dose experiments are necessary for further application of EPZ005687 in vivo.

EZH2 is important in cell cycle progression, cell proliferation, differentiation, apoptosis, DNA damage repair, and stem cell fate determination; loss of EZH2 leads to early embryonic lethality [36]. And the role of EZH2 in regulating oxidative stress is conflictive in different systems, for example, Yu et al. reported that inhibition of EZH2 against LPS-induced oxidative response in sepsis [37], whereas decreased EZH2 resulted in over-expression of a proapoptotic gene Bim and activation of oxidative reaction in erythroid cells [13]. The present study shows that inhibition of EZH2 by EPZ005687 reversed transcriptional repression of SOD1 in lung and delayed PAH progression in mouse TAC model. Given the diversity of EZH2 functions in regulation of oxidative reaction, a lineage or organ-specific EZH2 knockout or transgenic model might be more convincible. But, our results still provide some clues about pharmacologic targeting EZH2 in protection against PAH induced by TAC.

## 5. Conclusion

To sum up, increased EZH2 expression contributes to the progression of TAC-induced PAH by simulating ROS production and inhibiting SOD1 expression. The specific inhibitor of EZH2, EPZ005687, might reverse this deterioration of TAC-induced PAH. Ideally, using both RNA-seq and ChIP-seq techniques, in different types of cells in the context of TAC-induced PAH, then verified by a lineage or organ-specific EZH2 knockout or transgenic model, would hopefully render a more solid decision to target EZH2 for drug development.

## Abbreviations

EZH2: Enhancer of zeste homolog 2
TAC: Transverse aortic constriction
PAH: Pulmonary arterial hypertension
DMSO: Dimethyl sulfoxide
RVSBP: Right ventricular systolic blood pressure
PABP: Pulmonary arterial systolic blood pressure
DHE: Dihydroethidium
Real-time PCR: Real-time polymerase chain reaction
ChIP: Chromatin immunoprecipitation
ROS: Reactive oxygen species
SOD1: Superoxide dismutase 1
HF: Heart failure
CVDs: Cardiovascular diseases
H3K27me3: Histone H3 lysine 27 trimethylation
HW: Heart weight
LW: Lung weight
BW: Body weight
LV: Left ventricle
RV: Right ventricle
NT: Nitrotyrosine.
